# Supplementary material for: Solving an Old Puzzle: Elucidation and Evaluation of the Binding Mode of Salvinorin A at the Kappa Opioid Receptor
Source: Molecules. 2023 Jan 11;28(2):718. doi: 10.3390/molecules28020718 (PMC9861206; doi:10.3390/molecules28020718)
Supplement: Supplementary file 1 [file molecules-28-00718-s001.zip › molecules-2025011-supplementary.pdf]

Supporting Materials

Solving an Old Puzzle: Elucidation and Evaluation of the Binding Mode of Salvinorin A at the Kappa Opioid Receptor

Kristina Puls and Gerhard Wolber*

Department of Biology, Chemistry and Pharmacy, Institute of Pharmacy, Freie Universität Berlin; Königin-Luise-Str. 2+4, 14195 Berlin, Germany

*Correspondence: gerhard.wolber@fu-berlin.de (G.W.), Tel.: +49-30-838-52686

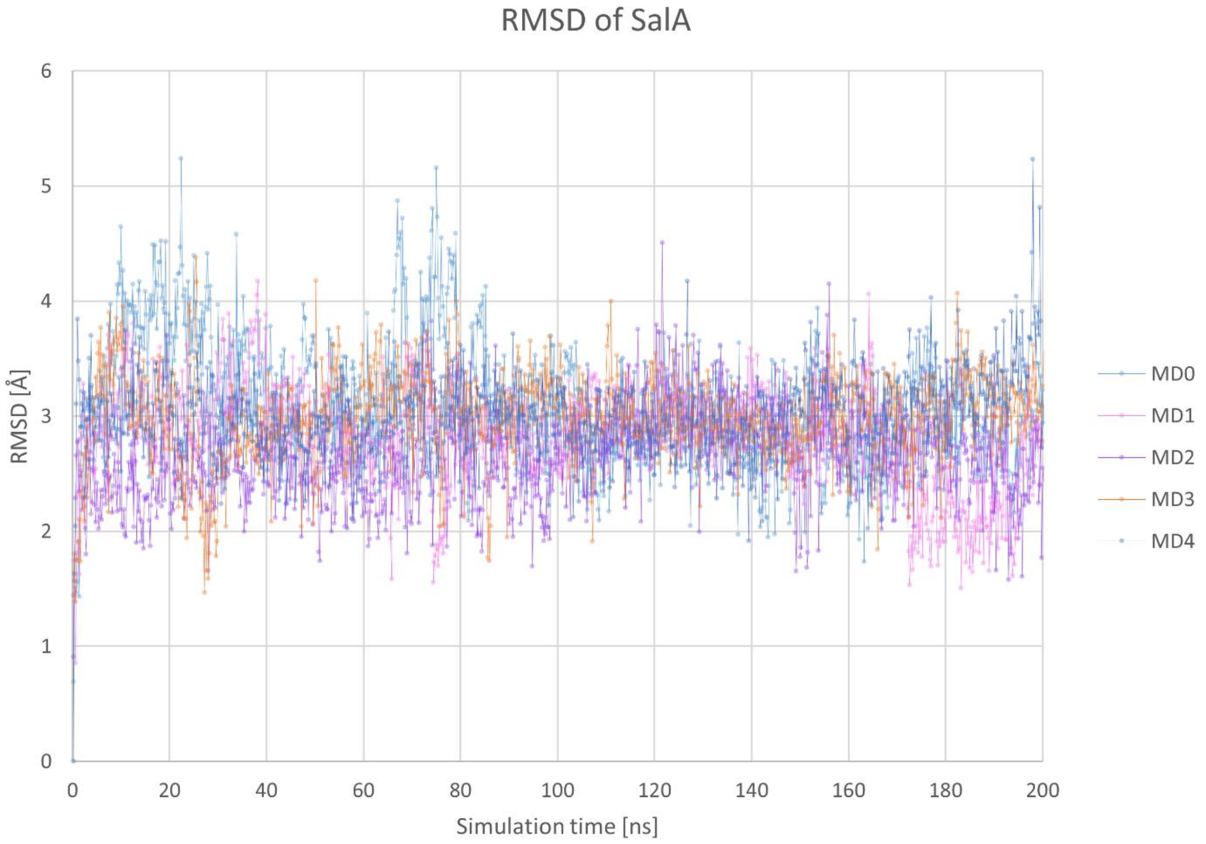

Figure S1. Root mean square deviation of SalA (**1**) in complex with the kappa opioid receptor (KOR) over the simulation time.

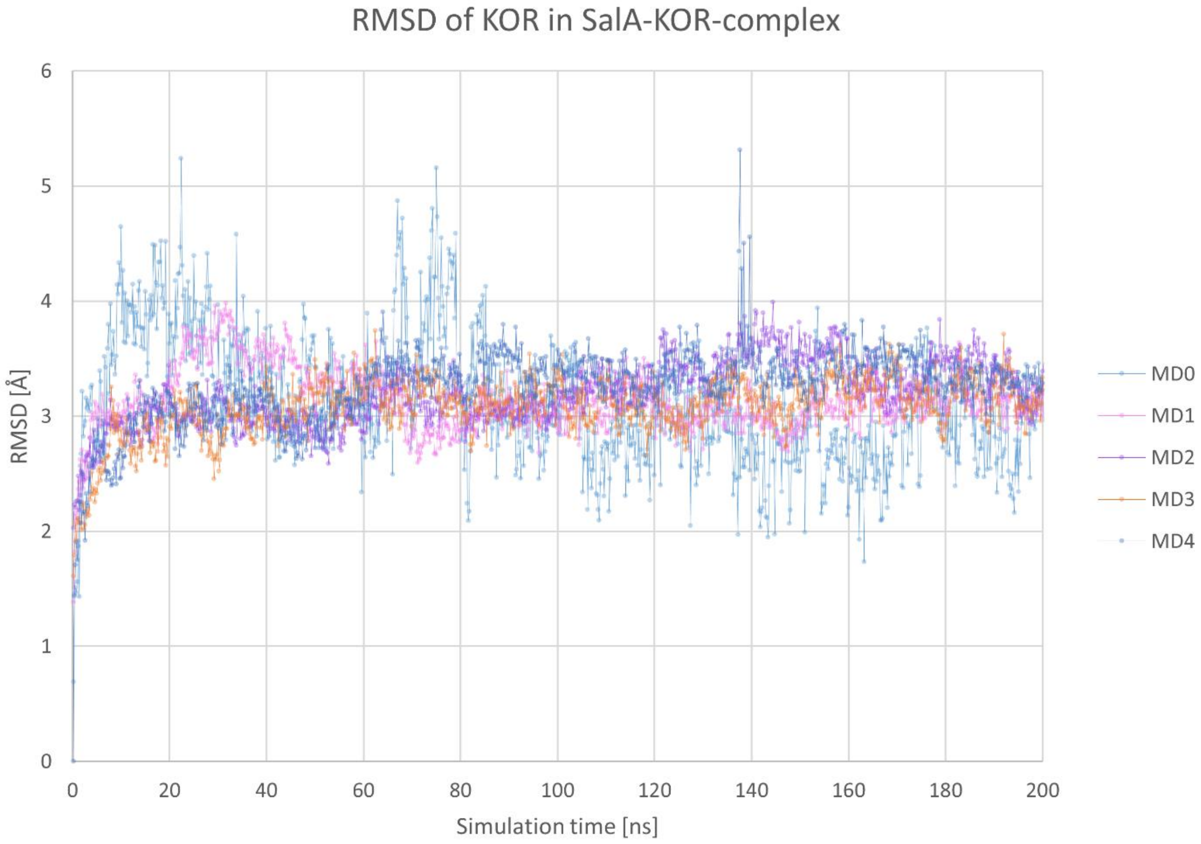

Figure S2. Root mean square deviation of the KOR backbone atoms in complex with SalA (**1**) over the simulation time.

Identity

	1.	2.	3.	4.
1. hKOR		53.5	57.4	50.3
2. hMOR	56.3		60.5	50.3
3.hDOR	56.3	56.2		50.0
4.hNOP	48.9	46.5	49.7	

Similarity

	1.	2.	3.	4.
1. hKOR		67.0	69.6	64.3
2. hMOR	70.5		70.7	63.8
3.hDOR	68.2	65.8		62.4
4.hNOP	62.6	59.0	62.1	

Figure S3. Sequence identity and similarity of the full sequence of KOR, MOR, DOR, and NOP.

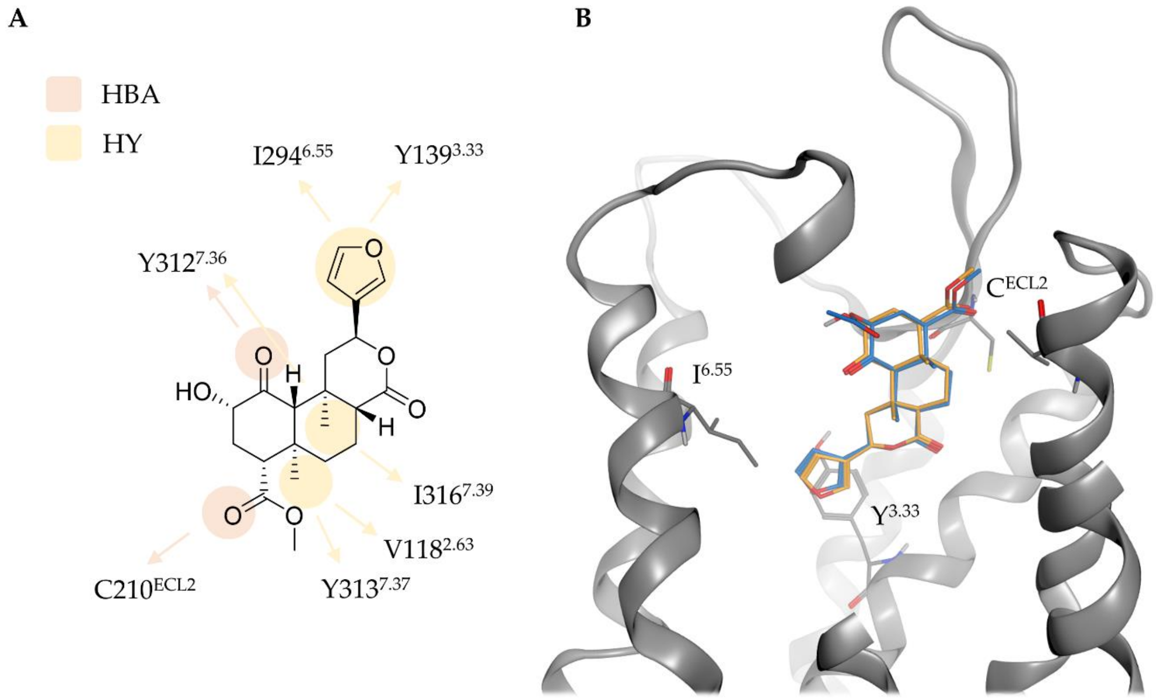

Figure S4. Protein-ligand interactions (A) and binding mode (B) of **2** (orange) at the KOR in comparison to SalA (blue). $Y^{3.33}$ denotes to $Y139^{3.33}$, C^{ECL2} to $C210^{ECL2}$, and $I^{6.55}$ to $I294^{6.55}$. Interactions types are abbreviated with HBA for hydrogen bond acceptor and HY for hydrophobic contact.

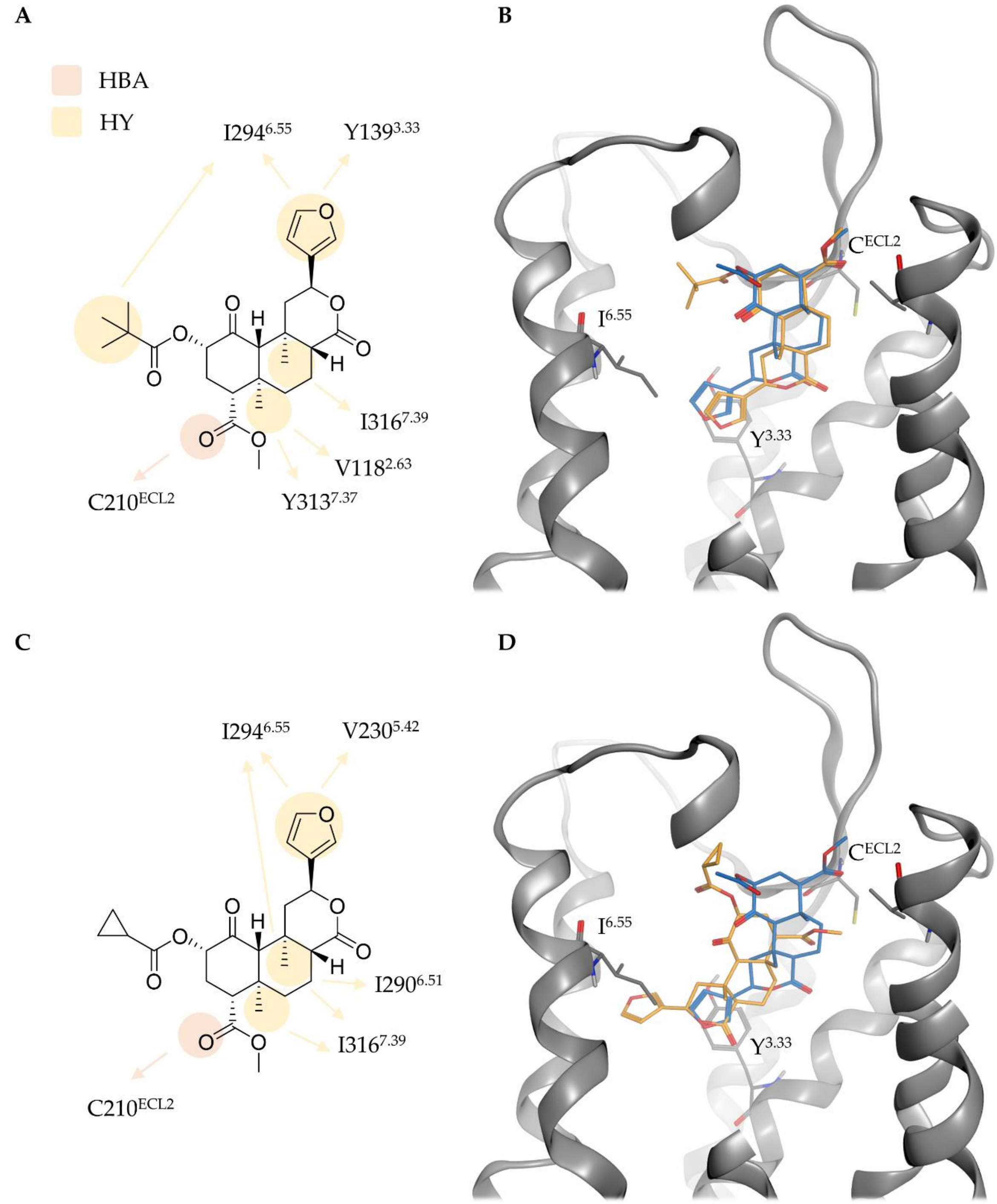

Figure S5. Protein-ligand interactions and binding mode of **3** (A, B, orange) and **4** (C, D, orange) at the KOR in comparison to SalA (blue). $Y^{3.33}$ denotes to $Y139^{3.33}$, C^{ECL2} to $C210^{ECL2}$, and $I^{6.55}$ to $I294^{6.55}$. Interactions types are abbreviated with HBA for hydrogen bond acceptor and HY for hydrophobic contact.

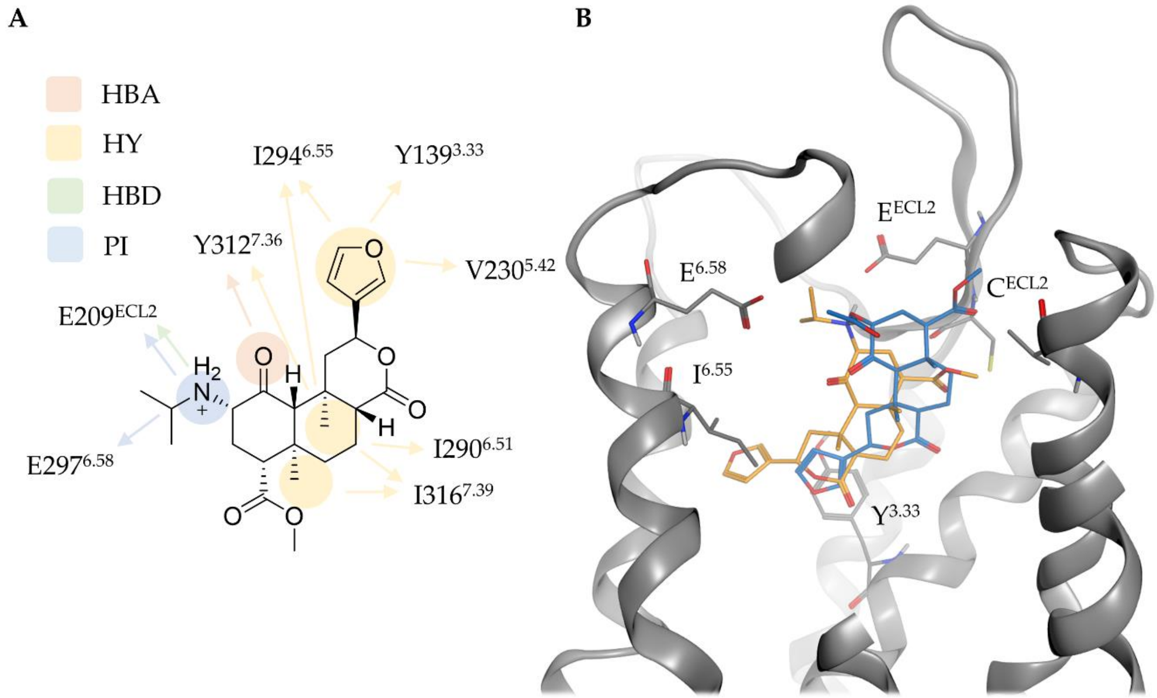

Figure S6. Protein-ligand interactions (A) and binding mode (B) of **5** (orange) at the KOR in comparison to SalA (blue). $Y^{3.33}$ denotes to $Y139^{3.33}$, C^{ECL2} to $C210^{ECL2}$, and $I^{6.55}$ to $I294^{6.55}$. Interactions types are abbreviated with HBA for hydrogen bond acceptor, HY for hydrophobic contact, HBD for hydrogen bond donor, and PI for positive charged interaction.

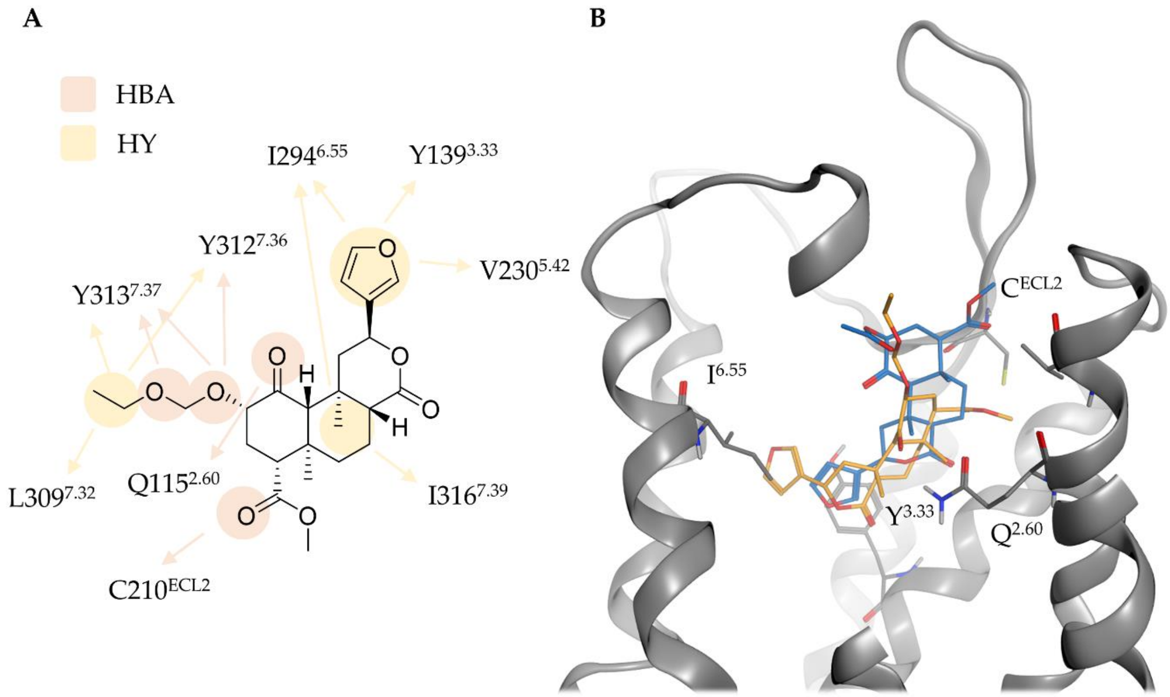

Figure S7. Protein-ligand interactions (A) and binding mode (B) of **6** (orange) at the KOR in comparison to SalA (blue). Y$^{3.33}$ denotes to Y139$^{3.33}$, C^{ECL2} to C210^{ECL2}, and I$^{6.55}$ to I294$^{6.55}$. Interactions types are abbreviated with HBA for hydrogen bond acceptor and HY for hydrophobic contact.

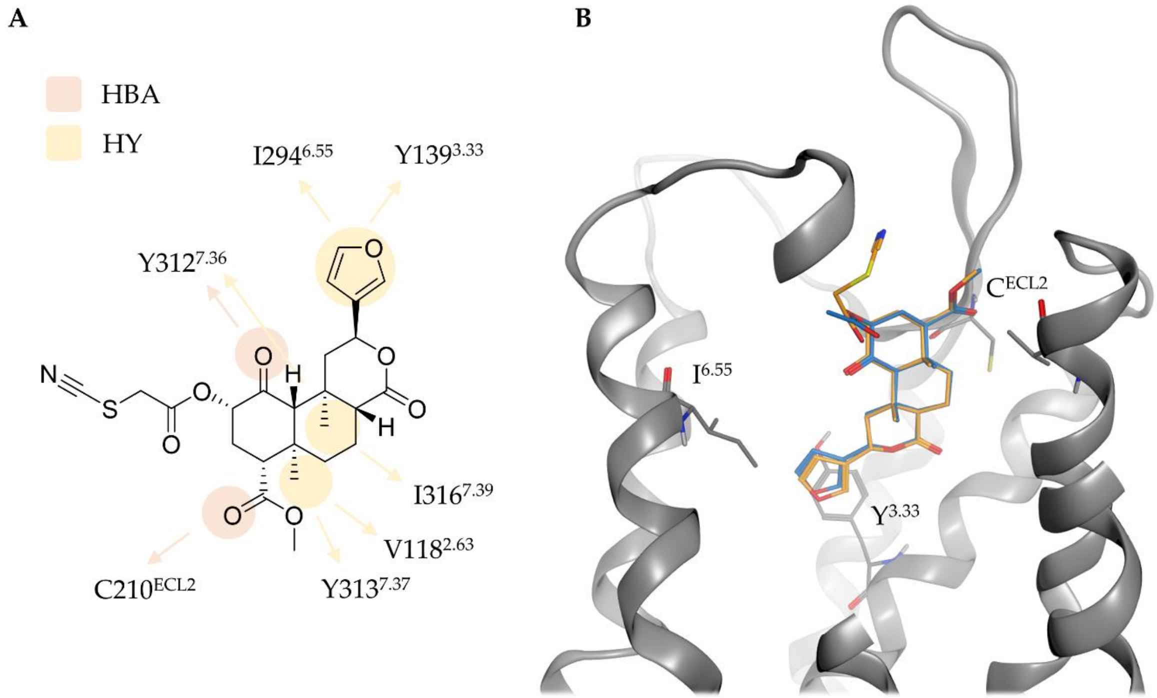

Figure S8. Protein-ligand interactions (A) and binding mode (B) of **7** (orange) at the KOR in comparison to SalA (blue). Y$^{3.33}$ denotes to Y139$^{3.33}$, C^{ECL2} to C210^{ECL2}, and I$^{6.55}$ to I294$^{6.55}$. Interactions types are abbreviated with HBA for hydrogen bond acceptor and HY for hydrophobic contact.

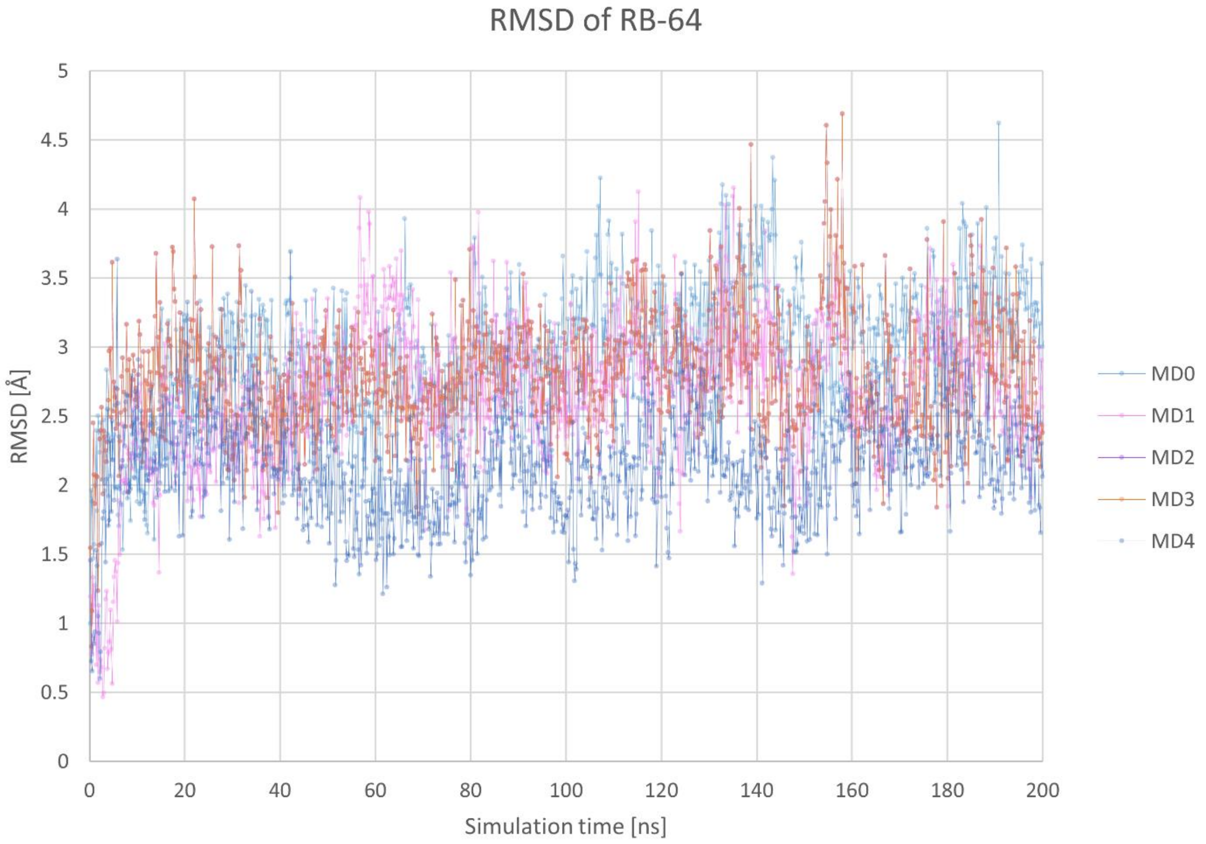

Figure S9. Root mean square deviation of RB-64 (**7**) in complex with the KOR over the simulation time.

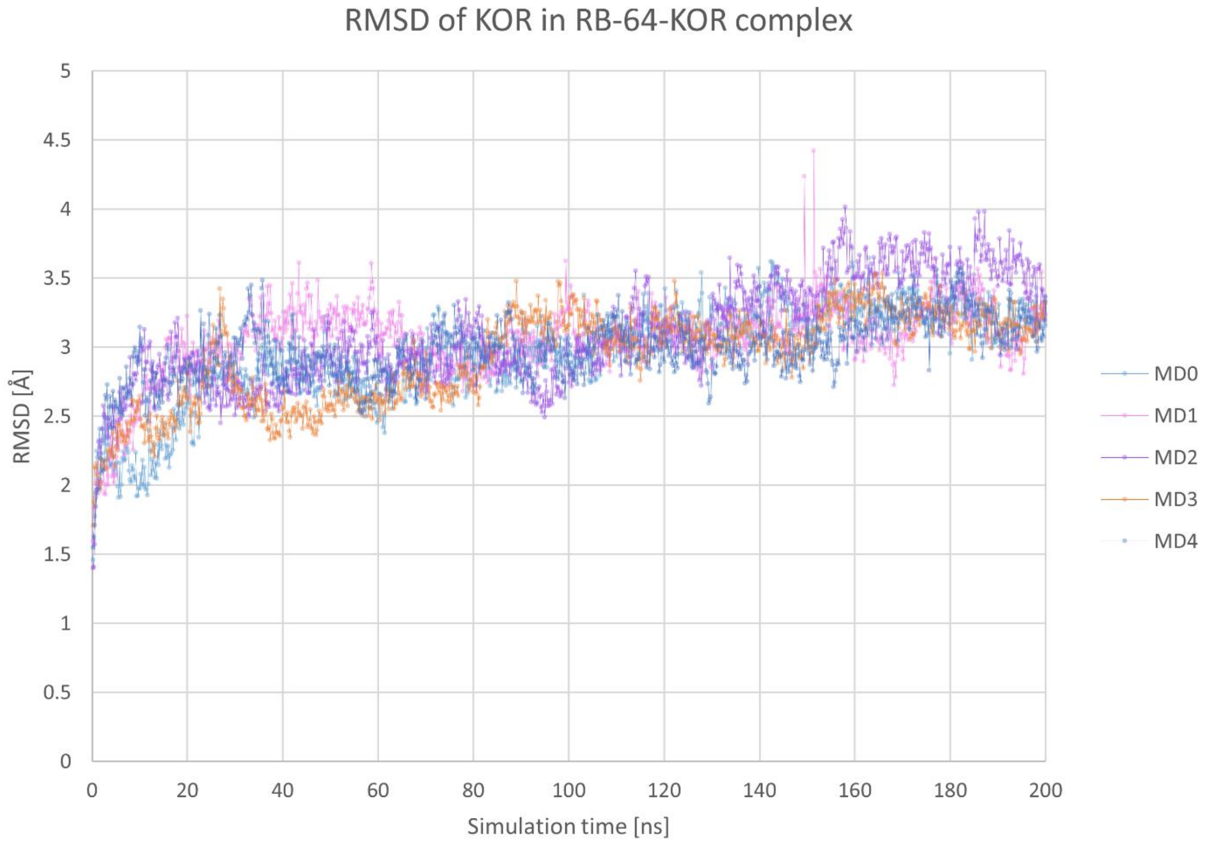

Figure S10. Root mean square deviation of the KOR backbone atoms in complex with RB-64 (**7**) over the simulation time.

Table S1. Energy calculations performed for the docking poses of SalA and RB64 bounded to the KOR.

<table>
<tr><th colspan="5">RBFE</th></tr>
<tr><th>Software</th><th>Calculation</th><th>ΔG complex (kcal/mol)</th><th>ΔG solvent (kcal/mol)</th><th>RBFE (kcal/mol)</th></tr>
<tr><td>Openfe [1]</td><td>Alchemical transformation of SalA into RB-64 bound to KOR</td><td>-27.273 ± 0.379</td><td>-25.059 ± 0.016</td><td>-2.214 ± 0.395</td></tr>
<tr><td>Schrödinger Ligand FEP [2,3]</td><td>Free energy perturbation turning RB-64 to SalA bound to KOR, respectively</td><td>-2.020 ± 0.202</td><td>-2.519 ± 0.214</td><td>0.499 ± 0.416</td></tr>
<tr><th colspan="5">ABFE</th></tr>
<tr><th>Software</th><th>Calculation</th><th>ABFE (kcal/mol)</th><th colspan="2">Difference in ABFE (kcal/mol)</th></tr>
<tr><td rowspan="2">YANK [4]</td><td>Binding free energy of SalA bound to KOR</td><td>-16.637 ± 0.961</td><td rowspan="2" colspan="2">∣ABFE(SalA)∣ – ∣ABFE(RB-64)∣

= -3.817 ± 2.03</td></tr>
<tr><td>Binding free energy of RB-64 bound to KOR</td><td>-20.454 ± 1.069</td></tr>
</table>

The abbreviations RBFE and ABFE refer to relative and absolute binding free energy. All methods predicted the binding of RB-64 to the active state KOR crystal structure (PDB-ID 6B73 [5]) favorable over the binding of SalA. The negative RBFE value in the case of openfe, where SalA is alchemically transformed into Rb-64, indicates that the latter (Rb-64) is favored over the first (SalA). The positive RBFE value in the case of Schrödinger Ligand FEP, where RB-64 is transformed into SalA, indicates the favorable binding of the first (RB-64) over the latter (SalA). In the case of YANK the absolute binding free energy of the two complexes, SalA or RB-64 bound to KOR, was calculated and compared. The more negative value for the RB-64 bound complex indicates an energetic improvement of the RB-64 bound state over the SalA-bound state.

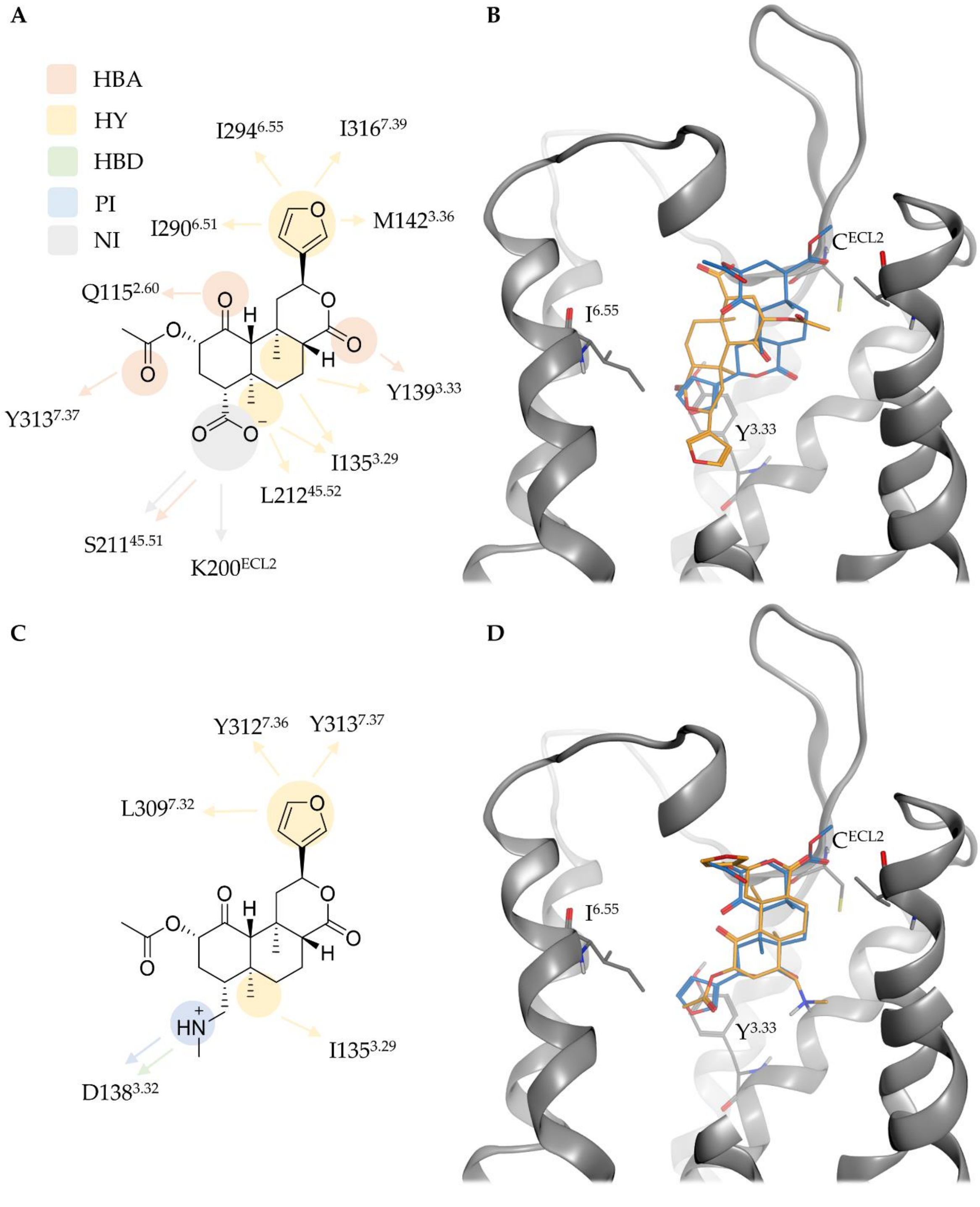

Figure S11. Protein-ligand interactions and binding mode of **8** (A, B, orange) and **9** (C, D, orange) at the KOR in comparison to SalA (blue). **8** is shifted in the binding side and shows an alternative Y139$^{3.33}$, C^{ECL2} to C210^{ECL2}, and I$^{6.55}$ to I294$^{6.55}$. Interactions types are abbreviated with HBA for hydrogen scaffold orientation while **9** shows a reversed orientation compared to SalA. Y$^{3.33}$ denotes to bond acceptor, HY for hydrophobic contact, HBD for hydrogen bond donor, NI for negative charged interaction, and PI for positive charged interaction.

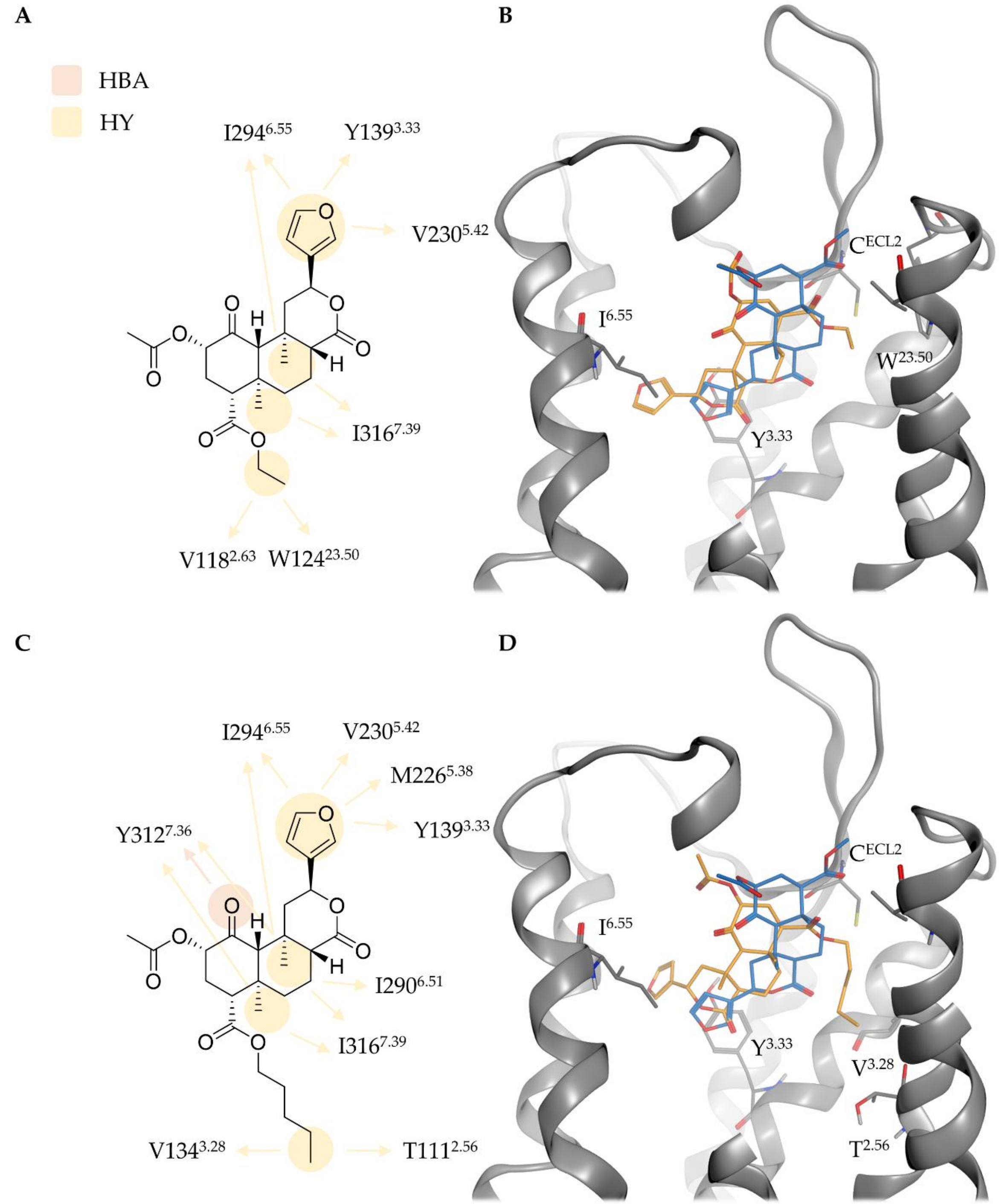

Figure S12. Protein-ligand interactions and binding mode of **10** (A, B, orange) and **11** (C, D, orange) at the KOR in comparison to SalA (blue). Y$^{3.33}$ denotes to Y139$^{3.33}$, C^{ECL2} to C210^{ECL2}, and I$^{6.55}$ to I294$^{6.55}$. Interactions types are abbreviated with HBA for hydrogen bond acceptor and HY for hydrophobic contact.

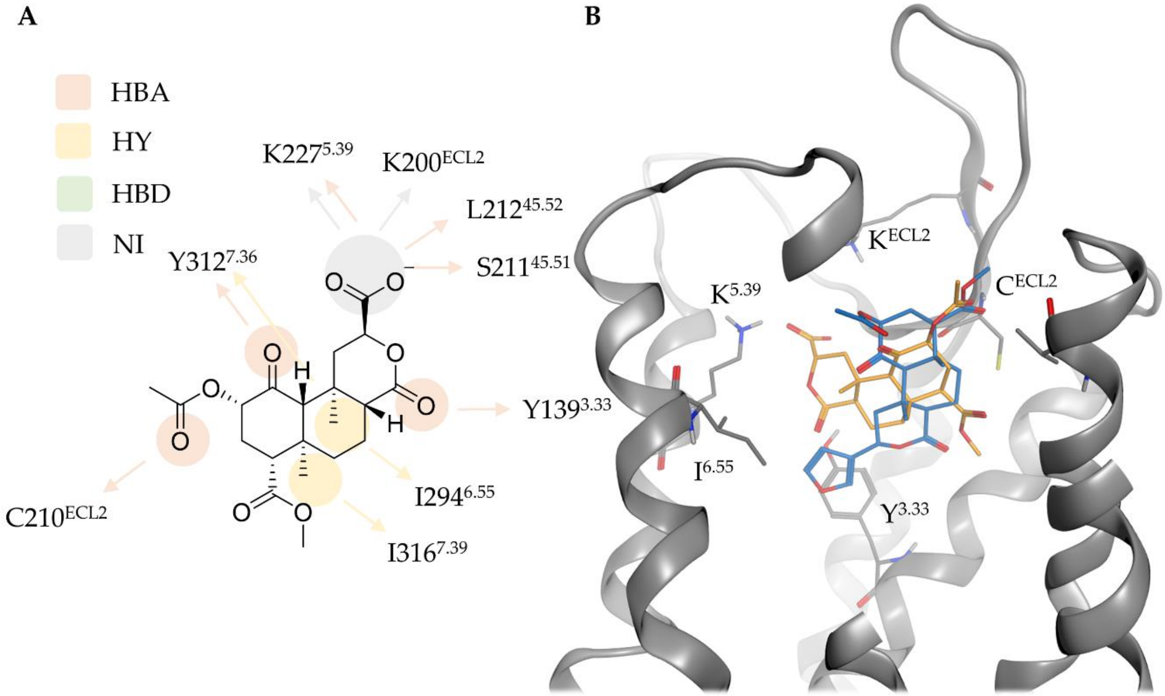

Figure S13. Protein-ligand interactions (A) and binding mode (B) of **13** (orange) at the KOR in comparison to SalA (blue). $Y^{3.33}$ denotes to $Y139^{3.33}$, C^{ECL2} to $C210^{ECL2}$, and $I^{6.55}$ to $I294^{6.55}$. Interactions types are abbreviated with HBA for hydrogen bond acceptor, HY for hydrophobic contact, HBD for hydrogen bond donor, and NI for negative charged interaction.

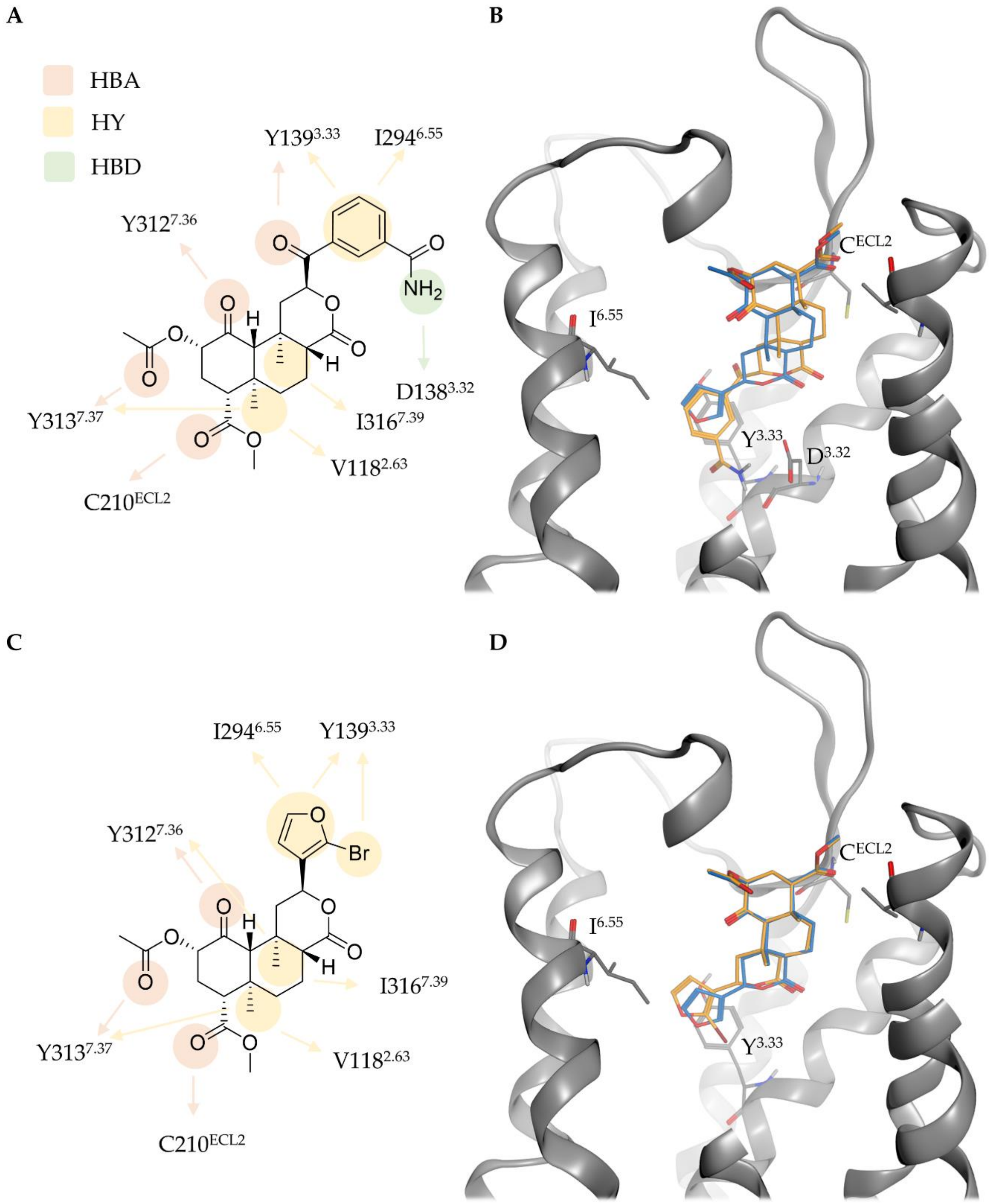

Figure S14. Protein-ligand interactions and binding mode of **14** (A, B, orange) and **15** (C, D, orange) at the KOR in comparison to SalA (blue). $Y^{3.33}$ denotes to $Y139^{3.33}$, C^{ECL2} to $C210^{ECL2}$, and $I^{6.55}$ to $I294^{6.55}$. Interactions types are abbreviated with HBA for hydrogen bond acceptor and HY for hydrophobic contact.

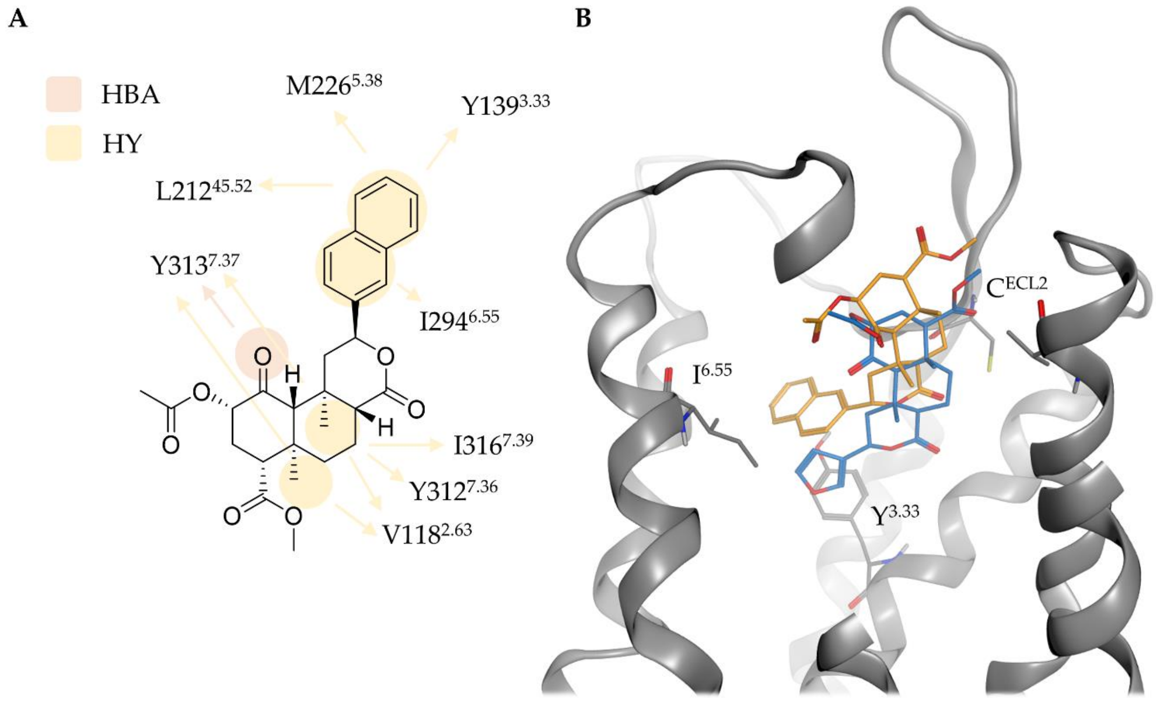

Figure S15. Protein-ligand interactions (A) and binding mode (B) of **16** (orange) at the KOR in comparison to SalA (blue). $Y^{3.33}$ denotes to $Y139^{3.33}$, C^{ECL2} to $C210^{ECL2}$, and $I^{6.55}$ to $I294^{6.55}$. Interactions types are abbreviated with HBA for hydrogen bond acceptor and HY for hydrophobic contact.

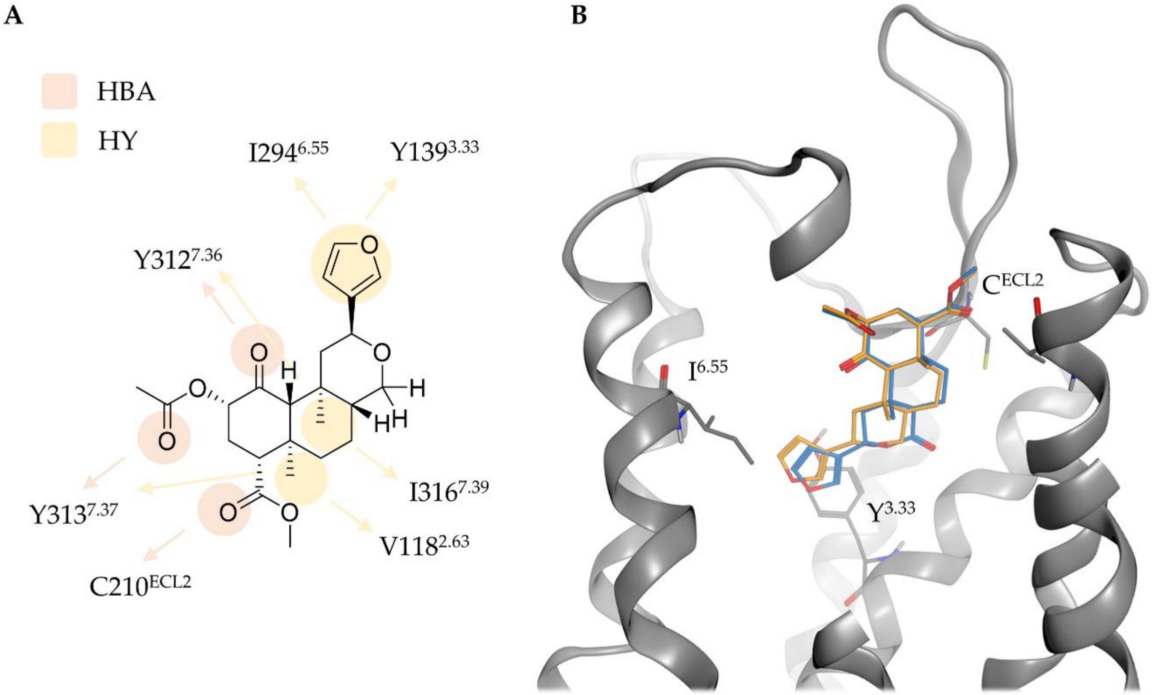

Figure S16. Protein-ligand interactions (A) and binding mode (B) of **17** (orange) at the KOR in comparison to SalA (blue). $Y^{3.33}$ denotes to $Y139^{3.33}$, C^{ECL2} to $C210^{ECL2}$, and $I^{6.55}$ to $I294^{6.55}$. Interactions types are abbreviated with HBA for hydrogen bond acceptor and HY for hydrophobic contact.

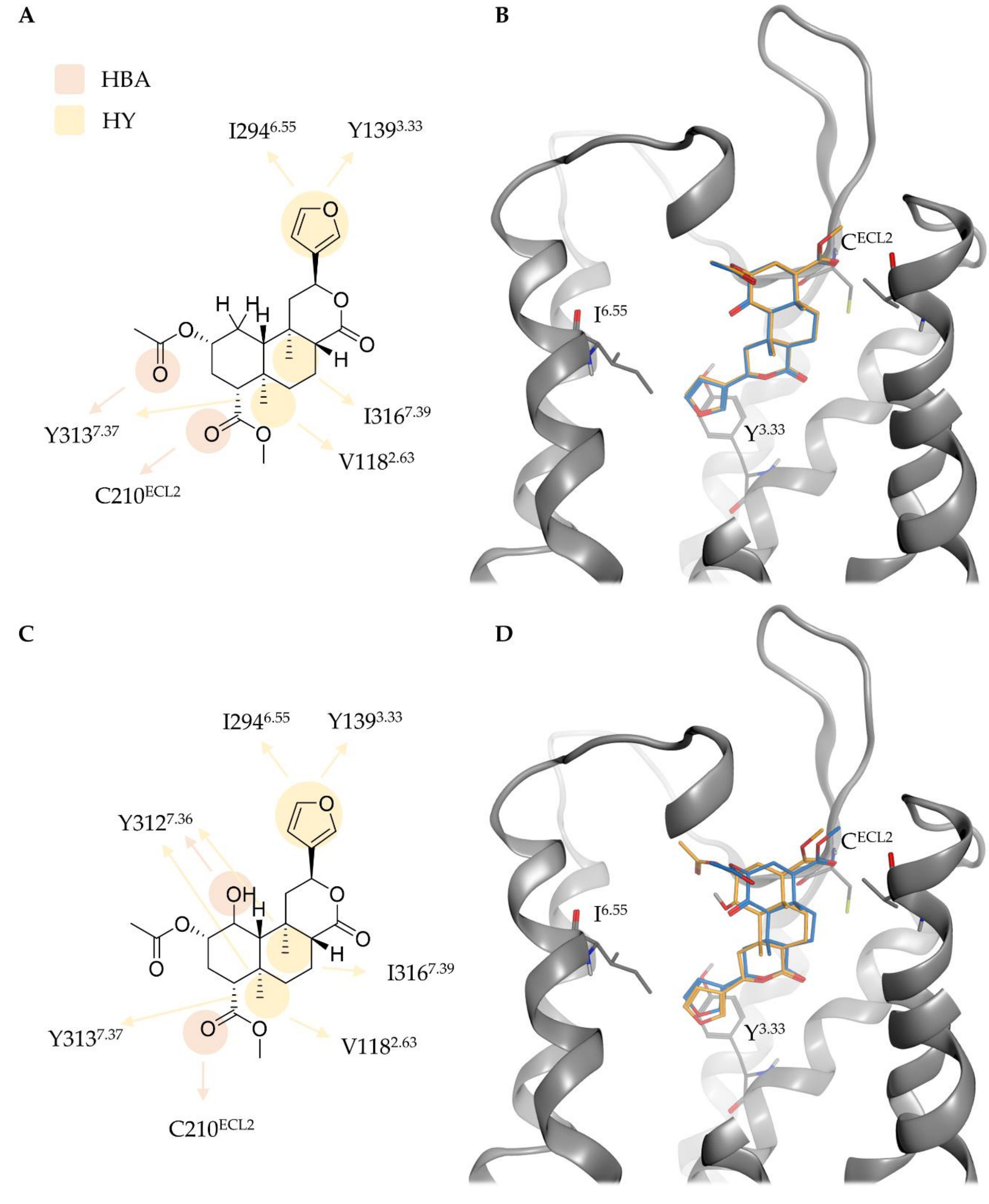

Figure S17. Protein-ligand interactions and binding mode of **18** (A, B, orange) and **19** (C, D, orange) at the KOR in comparison to SalA (blue Y$^{3.33}$ denotes to Y139$^{3.33}$, C^{ECL2} to C210^{ECL2}, and I$^{6.55}$ to I294$^{6.55}$. Interactions types are abbreviated with HBA for hydrogen bond acceptor and HY for hydrophobic contact.

References:

1. Gowers, R.J.; Alibay, I.; Swenson, D.W.H.; Henry, M.M. *Open Free Energy (openfe)*, version 0.21; 2022.
2. D E Shaw Research. *Desmond Molecular Dynamics System*, 2022-1; D E Shaw Research: New York, NY, USA, 2022.
3. Bowers, K.J.; Chow, D.E.; Xu, H.; Dror, R.O.; Eastwood, M.P.; Gregersen, B.A.; Klepeis, J.L.; Kolossvary, I.; Moraes, M.A.; Sacerdoti, F.D.; et al. Scalable algorithms for molecular dynamics simulations on commodity clusters. In Proceedings of the Proceedings of the ACM/IEEE Conference on Supercomputing (SC06), Tampa, FL, USA, 11-17 November 2006.
4. Rizzi A; Grinaway P.B.; Parton D.L.; Shirts M.R.; Wang K.; Eastman P.; Friedrichs M.; Pande V.S.; Branson K.; Mobley D.L.; et al. YANK: A GPU-accelerated platform for alchemical free energy calculations. http://getyank.org/latest/, accessed on December 1, 2022.
5. Che, T.; Majumdar, S.; Zaidi, S.A.; Ondachi, P.; McCorvy, J.D.; Wang, S.; Mosier, P.D.; Uprety, R.; Vardy, E.; Krumm, B.E.; et al. Structure of the nanobody-stabilized active state of the kappa opioid receptor. Cell **2018**, 172, 55-67.e15, doi:10.1016/j.cell.2017.12.011.
